# Impact of a tailored-care education programme on maternal and neonatal outcomes in pregnant women with gestational diabetes: a randomized controlled trial

**DOI:** 10.11604/pamj.2022.43.128.34084

**Published:** 2022-11-08

**Authors:** Sihem Chahed, Latifa Lassouad, Maha Dardouri, Ali Mtiraoui, Amel Maaroufi, Hedi Khairi

**Affiliations:** 1 Université de Sousse, Faculté de Médecine de Sousse, Laboratoire de Recherche LR12ES03 “Qualité des Soins et Management des Services de Santé Maternelle”, Sousse, Tunisia,; 2 Université de Monastir, Ecole Supérieure des Sciences et Techniques de la Santé de Monastir, Monastir, Tunisia,; 3 Hôpital Universitaire Farhat Hached, Service de Gynécologie-Obstétrique, Sousse, Tunisia,; 4 Hôpital Universitaire Farhat Hached, Service d´Endocrinologie et Diabétologie, Sousse, Tunisia

**Keywords:** Gestational diabetes, pregnant women, patient education, midwifery care

## Abstract

**Introduction:**

patient education is a key component of positive pregnancy and childbearing experiences, especially in women with gestational diabetes (GDM). Scant studies assessed the impact of tailored self-care education on pregnancy outcomes in pregnant women with gestational diabetes mellitus (GDM). This study aimed to assess the effect of a tailored-care education programme on maternal and neonatal outcomes in pregnant women with GDM during pregnancy and at birth.

**Methods:**

this was a randomized controlled trial conducted in a university hospital in the centre of Tunisia, from October 2020 to May 2021. The intervention group (n=61) received a self-care education programme with the usual care plan for GDM, while the control group received only the usual care plan (n=60). This trial was registered in the Pan African Clinical Trials Registry under the registration number PACTR202106591503674.

**Results:**

at baseline, there was no significant difference between groups in terms of sociodemographic and clinical characteristics. The findings showed that the intervention significantly reduced maternal and neonatal hospitalizations (p=0.000), caesarean section (p=0.002), preterm labour (p=0.002), macrosomia (p=0.000), foetal distress (p=0.001), newborn respiratory complication (p=0.01) and hypoglycaemia (p=0.000).

**Conclusion:**

implementing a tailored-care education for pregnant women with GDM had a positive impact on mother and infant clinical outcomes. Midwives and endocrinologists should use this programme to reduce maternal and neonatal complications during and after pregnancy.

## Introduction

Gestational diabetes mellitus (GDM) is a severe but neglected threat to maternal and child health [1]. It is defined as any degree of hyperglycaemia that is recognized for the first time during pregnancy [2]. In 2019, it was estimated that 1 in 6 births worldwide was affected by GDM [1]. Most cases of hyperglycaemia in pregnancy were in low- and middle-income countries, due to limited access to maternal healthcare. A recent systematic review revealed that pregnant women in the Middle East and North Africa region are burdened with a substantial prevalence of GDM, particularly in the Gulf Cooperation Council and North African countries [3]. In Tunisia, it was estimated that 9.3% of pregnant women developed GDM [4].

This condition is associated with multiple adverse pregnancy outcomes. Women with GDM experience high blood pressure, large birth weight babies, obstructed labour, and caesarean section [1,5]. Infants of diabetic mothers are exposed to metabolic and hematologic disorders, respiratory distress, cardiac disorders, and neurologic impairment due to perinatal asphyxia and birth traumas [6]. Additionally, the mother and baby are both at subsequent high risk of type 2 diabetes [1]. Therefore, it is substantial for women with GDM to carefully control and monitor their glycemia with their healthcare provider to reduce the risk of complications.

It is established that an early diagnosis associated with an adapted treatment is not enough to guarantee an optimal GDM control [7,8]. There is a strong connection between screening, diagnosis, treatment, and education. Indeed, recent studies revealed that self-care education using information and communication technologies was effective for blood sugar control, GDM knowledge, and a healthy lifestyle [9-12]. Nevertheless, pregnant women with low socioeconomic status and from low- and middle-income countries often have limited access to e-health programmes [1]. Besides, evidence showed that healthcare professionals and family support are key elements to empower women with GDM. Therefore, it is crucial to implement face-to-face education programmes for this population. According to the literature, different interventions were carried out to manage GDM [9,13-18]. Most of them were information technology (IT) programmes such as mobile health using smartphone applications X^18^,^19^], educational digital optical disc (DVD) [11], and web-based programmes [9,17,18]. Few studies implemented self-care education programmes for women with GDM. An Iranian clinical trial reported that self-care education had a positive effect on 2-h postprandial plasma glucose, APGAR scores, and neonatal hospitalization [14]. Another Iranian trial revealed that self-care education for pregnant women with GDM reduced caesarean section and macrosomia [15].

An Egyptian quasi-experimental study showed that self-care education was effective for GDM knowledge, attitudes, and self-care behaviour, and significantly reduced preterm and caesarean section [16]. The impact of self-care education on significant neonatal outcomes such as neonatal hypoglycaemia, foetal distress, birth defect, respiratory complications, and intra-uterine foetal death was poorly addressed. Besides, scant information exists about the effect of this programme on blood glucose control before birth and GDM-related hospitalization in low-middle income countries, such as Tunisia. Therefore, this study aimed to assess the effectiveness of a tailored self-care education programme on maternal and neonatal outcomes in pregnant women with GDM during pregnancy and at birth.

The hypothesis was that women with GDM who received the tailored self-care educational programme will develop fewer maternal and neonatal complications during pregnancy and at birth in comparison with the control group who received usual care education.

## Methods

**Study design:** this was a randomized controlled trial carried out in the Department of Endocrinology and the Department of Gynaecology at a University Hospital in the centre of Tunisia. The trial period was 8 months, from October 2020 to May 2021.

**Study population:** the target population was pregnant women with a diagnosed GDM by the obstetrician-gynaecologist in the first trimester, aged 19 to 44 years. Patients with chronic diabetes mellitus or other chronic diseases were not included. Participants were recruited from the endocrinology and gynaecology departments. The oral glucose tolerance test was used from 24 to 28 weeks for GDM screening in patients without any risk factors, such as advanced maternal age (≥35 years) [20], overweight or obesity, personal and family history of any form of diabetes [21]. In patients with risk factors of GDM, fasting blood glucose was used for GDM screening from the beginning of pregnancy up to 24 weeks.

**Sample size:** the sample size was calculated using the BiostaTGV with a power 1 - β = 0.8, two-tail α = 0.05, and a reduction with 0.6 in the mean of fasting blood glucose in the intervention group at follow-up. The required sample size was 72 pregnant women with GDM (36 women in each group). Considering a 20% of dropout and failure to consent, 50 participants in each group are needed.

**Figure 1:**
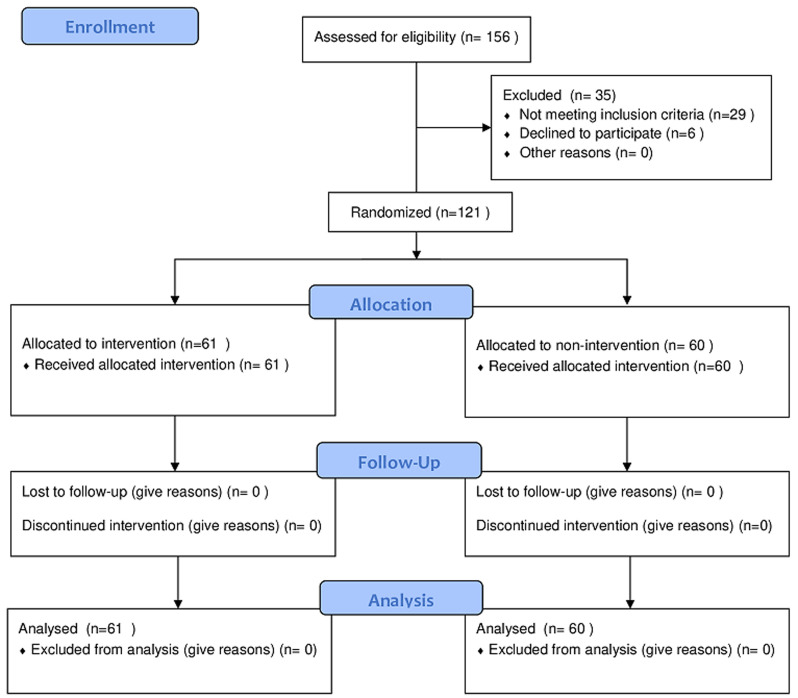
patients´ recruitment procedure

**Recruitment procedure:** Figure 1 shows the patients´ recruitment procedure. One hundred and fifty-six pregnant women who visited the gynaecology and the endocrinology departments in the first trimester of pregnancy were assessed for eligibility. Thirty-five women were excluded for the following reasons: 29 did not meet inclusion criteria, and 6 declined to participate. One hundred twenty-one were randomly allocated to the intervention and control groups by the principal investigator using sealed opaque envelopes. Each participant had an identification number (ID) (from 1 to 121) allocated by the research assistant. This latter submitted each ID in an individual sealed opaque envelope. The principal investigator randomly chooses from the envelopes to allocate the participant in the intervention or the control group. Using a coin, the first participant was allocated to the intervention group, the second to the control group, and the third to the intervention group, etc. The experimental group (n = 61) received an education programme according to a pre-established programme based on self-care with the usual care plan for GDM. The control group (n = 60) received only the usual care plan for GDM.

**Tailored self-care education programme:** the intervention aimed to enhance the self-capacity of women in GDM monitoring and management on daily basis through understanding the dietetic and hygiene measures. Self-care education was delivered by a multidisciplinary team composed of a midwife (principal investigator), an endocrinologist, a nurse, and a dietician. The intervention was carried out in the conference room of the Endocrinology Department of Farhat Hashed University Hospital for six consecutive days. The content was developed by the research team. It was based on international recommendations [1,22]. It included the pathophysiology of GDM and its complications, the technique of injecting insulin by syringe, the equipment and technique of self-monitoring by glucometer, and the nutrition and diet of patients with GDM. The nutritional and dietary model was based on the recommendations of the British Diabetic Association [22]. These recommendations included eating healthier carbohydrates like whole-grain bread, brown rice, vegetables (e.g. 1 sweet potato per day) 5 types of fruits a day (150ml of fruit juice, one handful of grapes, seven strawberries, two tinned pineapple rings), cutting down sugar and replacing it with low or zero-calorie sweeteners, perfecting portion sizes (2 heaped tablespoons of cooked rice, 3 tablespoons of breakfast cereal, 3 heaped tablespoons of boiled pasta or cooked noodles, 60g of reduced or low-fat cream cheese, 125g of low-sugar yogurt), understanding the glycaemic index, eating more fish (a portion of 140g per day). The utilization of the educational programme was described in detail in Table 1. On the first day, the physician and the midwife conducted an individual preliminary interview with participants to discuss their current health status, to present the programme, to define their objectives, and to identify their expectations.

**Table 1:** self-care education programme

Session 1 (one hour)	Educators	Monday	Tuesday	Wednesday	Thursday	Friday
		Midwife, endocrinologist, and dietitian	Midwife and nurse	Midwife and dietitian	Midwife and nurse	Midwife, endocrinolo-gist, and dietician
	**Content**	Individuals interviews; initial discussion of knowledge and eating habits; presentation of the week's programme	Insulin injection technique: injection's sites, types of insulin	The diet of a patient with gestational diabetes and dietary equivalents	Self-monitoring urine analysis capillary glycemia self-monitoring notebook	Evaluation of achievements questions and answers
**Session 2 (one hour)**	**Educators**	Endocrinologist	Endocrinologist	Endocrinologist	Endocrinologist	
	**Content**	General facts about gestational diabetes: what is gestational diabetes? What are the complications of gestational diabetes?	Severe complications of gestational diabetes, signs, causes, treatment, and prevention measures	Body hygiene importance of physical activity	Pregnancy and gestational diabetes: risks; planning and monitoring	

The intervention group was divided into 8 subgroups of 6 to 8 women homogeneous in term of gestational age. This group division helped to build strong communication between participants in the same gestational age and to exchange their experiences. For example, group one was composed of women with 17 to 20 weeks of pregnancy (n=19). It was then divided into 2 subgroups of 6 and one subgroup of 7 participants.

Women in the intervention group self-monitor their blood glucose control daily at home using a glucometer provided by the research team. This glucometer helped women to test their blood glucose on daily basis. Basing on the test results, they can self-regulate their diet according to the recommendations provided during the education program. The participants received a brief reminder of the education programme during GDM consultation. The women were followed-up monthly during GDM consultations to ensure compliance with the education program using the daily booklet of GDM self-monitoring. In a glycaemic cycle using glucometer (test before meals and 2 hours after meals 3 times per day), if the fasting blood glucose was ≥5.3 mmol/L and/or the blood glucose 2 hours after meal was ≥6.7 mmol/L, this means that the woman did not respect the recommendations.

**Usual care plan:** at the Obstetrics and gynaecology Department of Farhat Hashed University Hospital, women with gestational diabetes systematically and regularly benefit from specialized prenatal care plans with a monitoring protocol that meets international standards, to optimize the management of the pregnancy.

This care plan consisted of: a first consultation with the endocrinologist for general information about healthy eating, diet, and insulin therapy technique if needed, as well as the aims and the benefits of the follow-up consultations; providing all patients with usual care in the high-risk pregnancy unit of the maternity and neonatal centre of the hospital; an obstetrical consultation every 15 days for all women with GDM from 32 weeks until hospitalization; hospitalization from 38 weeks allows closer monitoring of the patient and a recording of the foetal heart rate twice daily. Pre-aesthetic consultation is planned for hospitalized patients and corticosteroid therapy is prescribed when indicated. A concertation with the neonatologist is systematic before any decision of planned caesarean section. All new-borns were examined at birth by a paediatrician.

**Data collection:** sociodemographic data, family history with diabetes mellitus, personal history (GDM, fetal macrosomia, intrauterine fetal demise (IUFD), pregnancy toxemia, prematurity) and clinical information related to the current pregnancy and GDM were collected from patients´ records using a data collection sheet. The primary outcomes were the number of hospitalizations due to GDM, the number of hospitalization days, and blood glucose control. This later was recorded based on the number of blood glucose cycles during hospitalizations, fasting blood glucose, and hypoglycaemia. A glycemic cycle consists of repetitive blood glucose tests 6 times a day: before meals and 2 hours after meals 3 times per day.

The secondary outcomes were the birth term (premature ≤36 weeks, full-term ≥37 weeks), the birth mode (vaginal, caesarean section), and the neonatal outcomes at birth (foetal distress, Appearance, Pulse, Grimace, Activity and Respiration (APGAR) score at 1 and 5 minutes, birth weight, hospitalization, respiratory complications, glycemia, foetal infection, birth defect, and intra-uterine foetal death).

**Statistical analysis:** data were analysed using the IBM SPSS Statistics version 20.0. The normal distribution of continuous variables was assessed using the Kolmogorov-Smirnov test. If the p value was significant (p≤0.05) the hypothesis that the respective distribution is normal was rejected. Continuous variables were described as means and standard deviation. The Kolmogorov-Smirnov test showed that continuous variables in this study were not normally distributed. Therefore, we used Mann-Whitney to assess the difference between groups. Categorical variables (age group, socioeconomic status, family, and personal histories, etc.) were described as frequencies and percentages. To compare the difference between groups in terms of categorical variables, Chi square test was used. A significance level of 0.05 was considered.

**Ethics:** this study was approved by a local research ethics committee under the reference code CEFMS 79/2021. It was registered to the Pan African Clinical Trials Registry under the registration number PACTR202106591503674. Participants were informed and they signed written consent before their enrolment in the study. The obtained data were confidential and anonymous.

## Results

Table 2 shows that there was no significant difference between the intervention and the control groups at baseline in terms of sociodemographic and clinical data. In the intervention group, the mean term of the current pregnancy at GDM diagnosis was 15.82 ± 1.86 weeks of gestation, and in the control group, it was 15.41 ± 2.20. The mean of gesture in the intervention group was 2.51 ± 1.44 and in the control group 2.82 ± 1.58.

**Table 2:** sociodemographic and clinical data of participants in the intervention and the control groups at baseline

Variables	IG (n=61) n (%)	CG (n=60) n (%)	X^2^	p
**Age of the participant**			**0.199**	**0.65**
20-34 years	37 (60.7)	34 (56.7)		
35-45 years	24 (39.3)	26 (43.3)		
**Socioeconomic status**			**0.017**	**0.89**
Low	20 (32.8)	19 (31.7)		
Medium-high	41 (67.2)	41 (68.3)		
**Educational level**			**0.406**	**0.52**
Illiterate-primary	20 (32.8)	23 (38.3)		
Secondary-university	41 (67.2)	37 (61.7)		
**Family history**				
**Diabetes mellitus type 1**			**0.109**	**0.74**
Yes	41 (67.2)	42 (70)		
No	20 (32.8)	18 (30)		
**Diabetes mellitus type 2**			**0.689**	**0.4**
Yes	30 (49.2)	25 (41.7)		
No	31 (50.8)	35 (58.3)		
**Personal history**				
**Gestational diabetes mellitus (GDM)**			**0.730**	**0.39**
Yes	16 (26.2)	20 (33.3)		
No	45 (73.8)	40 (66.7)		
**Foetal macrosomia**			**0.169**	**0.68**
Yes	13 (21.3)	11 (18.3)		
No	48 (78.7)	49 (81.7)		
**Pregnancy toxaemia**			**2.480**	**0.11**
Yes	11 (18)	5 (8.3)		
No	50 (82)	55 (81.7)		
**Intra uterine foetal death**			**0.325**	**0.57**
Yes	2 (3.3)	1 (1.7)		
No	59 (96.7)	59 (98.3)		
**Prematurity**			**0.058**	**0.8**
Yes	8 (13.1)	7 (11.7)		
No	53 (86.9)	53 (88.3)		
**Current pregnancy**				
**Body mass index**			**2.534**	**0.11**
Non-obese (≤30 Kg/m^2^)	29 (47.5)	20 (33.3)		
Obese (>30 Kg/m^2^)	32 (52.5)	40 (66.7)		
**Pregnancy toxaemia**			**1.99**	**0.15**
Yes	5 (8.2)	10 (16.7)		
No	56 (91.8)	50 (83.3)		

IG: intervention group; CG: control group

**Comparison of primary outcomes during pregnancy follow-up:** Table 3 shows that the number of hospitalizations due to GDM, the number of hospitalization days, and the number of blood glucose cycles during all hospitalizations were significantly higher in the control group in comparison with the intervention group (p= 0.000). At baseline, there was not a significant difference between groups in term of fasting blood glucose (p=0.07).

**Table 3:** comparison of primary outcomes between groups at baseline and follow-up

Variables	IG (n=61)	CG (n=60)	X^2^	U Mann-Whitney	p
Number of hospitalizations due to GDM during pregnancy (m ± SD)	1.02±0.12	2.38±0.49	-	18.5	0.000
Number of hospitalization days (m ± SD)	1.28±0.45	2.67±0.85	-	272	0.000
Number of blood glucose cycles during all hospitalizations (m ± SD)	1.31±0.56	5.07±2.007	-	46	0.000
Fasting blood glucose at baseline (m ± SD)	0.98±0.14	1.20±0.26	-	1497.5	0.07
Fasting blood glucose at follow-up (m ± SD)	0.92±0.09	1.15±0.26	-	979	0.000
Hypoglycaemia at baseline, n (%)	6 (9.8)	22 (36.7)	12.243	-	0.000
Hypoglycaemia at follow, n (%)	5 (8.2)	13 (21.7)	4.334	-	0.03

IG: intervention group; CG: control group; GDM: gestational diabetes mellitus

In the intervention group, the mean of fasting blood glucose was significantly reduced at follow-up (p=0.000). The rate of women with hypoglycaemia at admission and discharge was significantly higher in the control group than in the intervention group (respectively at admission: 36.7% and 9.8%, p=0.000; respectively at discharge: 21.7% and 8.2%, p=0.03).

**Comparison of secondary outcomes after pregnancy follow-up:** Table 4 reports that 3 women in the intervention group and 15 women in the control group had premature labour, with a significant difference (p=0.002). The rate of caesarean section in the control group was significantly higher than in the intervention group (p=0.002). Regarding new born health outcomes, there was a significant difference between groups in terms of foetal distress (p=0.001), APGAR score at 1 and 5 minutes (p=0.002; p=0.001, respectively), neonatal hospitalization (p=0.000), respiratory complication (p=0.01), and hypoglycaemia (p=0.000).

**Table 4:** comparison of secondary outcomes between groups at follow-up

Variables	IG (n=61) n (%)	CG (n=60) n (%)	X^2^	p
**Birth term**			**9.633**	**0.002**
Premature (≤36 weeks)	3 (4.9)	15 (25)		
Full-term (≥37 weeks)	58 (95.1)	45 (75)		
**Birth mode**			**9.285**	**0.002**
Vaginal	45 (68.9)	27 (45)		
Caesarean section	19 (31.1)	33 (55)		
**New-born weight at birth**			**31.017**	**0.000 ^a^**
<4 kg	61 (100%)	35 (58.3)		
≥4 kg	0 (0)	24 (41.7)		
**APGAR score at 1 mn**			**9.436**	**0.002**
<7	1 (1.6)	11 (18.3)		
≥7	60 (98.4)	49 (81.7)		
**APGAR score at 5 mn**			**9.885**	**0.001 ^a^**
<7	0 (0)	9 (15)		
≥7	61 (100)	51 (85)		
**Neonatal hospitalization**			**39.979**	**0.000**
Yes	9 (14.8)	43 (71.7)		
No	52 (85.2)	17 (28.3)		
**Neonatal respiratory complication**			**5.760**	**0.01**
Yes	6 (9.8)	16 (26.7)		
No	55 (90.2)	44 (73.3)		
**Foetal distress**			**11.81**	**0.001**
Yes	2 (3.3)	15 (25)		
No	59 (96.7)	45 (75)		
**Neonatal hypoglycaemia**			**18.745**	**0.000 ^a^**
Yes	0 (0)	16 (26.7)		
No	61 (100)	44 (73.3)		
**Maternal-foetal infection**			**1.069**	**0.3**
Yes	1 (1.6)	3 (5)		
No	60 (98.4)	57 (95)		
**Birth defect**			**4.206**	**0.05 ^a^**
Yes	0 (0)	4 (6.7)		
No	61 (100)	56 (93.3)		
**Intra uterine foetal death**			**1.025**	**0.49 ^a^**
Yes	0 (0)	1 (1.7)		
No	61 (100)	59 (98.3)		

a : Fisher test p-value; APGAR: appearance, pulse, grimace, activity and respiration; IG: intervention group; CG: control group

## Discussion

Education programmes for pregnant women with GDM aiming to strengthen self-care are needed [13]. The present study aimed to assess the effectiveness of a tailored-care education programme on maternal and neonatal outcomes in pregnant women with GDM. The findings showed that women who received a tailored-care education programme with a multidisciplinary team including a dietitian, a midwife, a nurse, and an endocrinologist had significantly a lower number of hospitalizations due to GDM, a lower rate of hypoglycaemia, premature labour, and caesarean section in comparison to the control group. Additionally, new-borns of women in the intervention group had significantly greater APGAR scores at 1 and 5 minutes, a normal birth weight, and less foetal distress, neonatal complications including neonatal hospitalization, respiratory complication, and hypoglycaemia in comparison to the control group.

The current findings are consistent with the results of the current literature. Mirghafourvand *et al*. found that self-care training on physical activity and nutrition reduced the rate of macrosomia and caesarean section in Iranian pregnant women with GDM [15]. Kolivand *et al*. reported that a self-care guide package delivered in face-to-face educational sessions had a positive effect on maternal self-efficacy and 2-h postprandial plasma glucose, APGAR scores, and neonatal hospitalization in Iranian pregnant women with GDM [14]. Ibrahim and Saber revealed that Egyptian pregnant women with GDM who received self-care education programmes had significantly better GDM knowledge, attitudes, and self-care behaviour and fewer neonatal complications including hypoglycaemia, macrosomia, and hospitalization at the neonatal intensive care unit immediately after birth [16]. In the same study, the authors found that a self-care education programme did not significantly reduce prematurity and respiratory distress. Likewise, Mirghafourvand *et al*. revealed that there were no significant differences between groups in terms of preterm labour [15].

What the current study adds is that a tailored-care education programme with a multidisciplinary team significantly reduced preterm labour, respiratory complications, foetal distress, neonatal hospitalization, macrosomia, and hypoglycaemia immediately after birth. However, there was no significant difference between the intervention and the control group in terms of foetal infection, birth defect, and intra-uterine foetal death. Regarding maternal complications, this intervention improved blood glucose control and reduced the rate of mother hospitalizations due to GDM, hypoglycaemia during pregnancy, vaginal specimen, glycosuria, and caesarean section in comparison to the control group.

**Limitations:** the present study had some limitations. First, double blinding was not possible to use. Second, this study was monocentric, which makes the results not generalizable. However, monocentric studies conserve the homogeneity of the sample. As well, it would be interesting if this study assessed the impact of tailored-care education on anxiety, depression, and stress in pregnant women with GDM.

**Implications for practice:** the current findings are beneficial for midwives and nurses practicing in women´s health centres. It is highly recommended to implement face-to-face and tailored-care education programmes for pregnant women with GDM from low- and middle-income countries. This fact can strengthen the connection between patients and healthcare providers and improve access to maternal and child healthcare centres. Tailored-care education programmes for pregnant women with GDM should be an urgent project of maternal and child health policy since it can improve healthcare coverage and reduce health inequalities in low- and middle-income countries. Besides, this intervention reduced hospitalization due to GDM, which helps to limit the overload of patients in the endocrinology department and the economic burden of GDM.

## Conclusion

The present study revealed that a tailored-care education programme with a multidisciplinary team including a midwife, a nurse, a dietitian, and an endocrinologist reduced mother hospitalizations due to GDM, hypoglycaemia during pregnancy, preterm labour, caesarean section, macrosomia, and neonatal complications including neonatal hospitalization, respiratory complication, foetal distress, and hypoglycaemia. The need for making tailored-care education programmes and maternal and child healthcare policy for pregnant women with GDM in low- and middle-income countries should be considered. This fact can improve healthcare coverage and reduce health inequalities in low- and middle-income countries. Tailored-care education help to limit the overload of patients in the endocrinology department and the economic burden of GDM.

### What is known about this topic

- Good antenatal care and patient education are crucial for a positive pregnancy and childbearing experience, particularly in pregnant women with gestational diabetes;
- Few studies implemented a tailored-care education programme and explore its effects on maternal and neonatal outcomes in low- and middle-income countries;
- Self-care education in pregnant women with gestational diabetes improved glycaemic control and APGAR scores and reduced caesarean section and neonatal hospitalizations.

### What this study adds

- Implementing a tailed self-care education for pregnant women with GDM had a positive and broad impact on mother and new born clinical outcomes;
- For mothers, it reduced mother hospitalizations due to GDM, hypoglycaemia during pregnancy, preterm labour, caesarean section;
- For babies, this intervention reduced the rate of macrosomia, respiratory complication, foetal distress, and hypoglycaemia.
